# Adapted Boolean network models for extracellular matrix formation

**DOI:** 10.1186/1752-0509-3-77

**Published:** 2009-07-21

**Authors:** Johannes Wollbold, René Huber, Dirk Pohlers, Dirk Koczan, Reinhard Guthke, Raimund W Kinne, Ulrike Gausmann

**Affiliations:** 1 Systems Biology/Bioinformatics, Leibniz Institute for Natural Product Research and Infection Biology – Hans Knöll Institute, Beutenbergstr. 11a, 07745 Jena, Germany; 2 Institute of Algebra, Technische Universität Dresden, Zellescher Weg 12-14, 01062 Dresden, Germany; 3 Experimental Rheumatology Unit, Department of Orthopaedics, University Hospital Jena, Friedrich Schiller University Jena, Klosterlausnitzer Str. 81, 07607 Eisenberg, Germany; 4 Institute of Clinical Chemistry, Hannover Medical School, Carl-Neuberg-Str. 1, 30625 Hannover, Germany; 5 Proteome Center Rostock, University of Rostock, Schillingallee 69, 18055 Rostock, Germany; 6 Genome Analysis, Leibniz Institute for Age Research – Fritz Lipmann Institute, Beutenbergstr.11, 07745 Jena, Germany

## Abstract

**Background:**

Due to the rapid data accumulation on pathogenesis and progression of chronic inflammation, there is an increasing demand for approaches to analyse the underlying regulatory networks. For example, rheumatoid arthritis (RA) is a chronic inflammatory disease, characterised by joint destruction and perpetuated by activated synovial fibroblasts (SFB). These abnormally express and/or secrete pro-inflammatory cytokines, collagens causing joint fibrosis, or tissue-degrading enzymes resulting in destruction of the extra-cellular matrix (ECM).

We applied three methods to analyse ECM regulation: data discretisation to filter out noise and to reduce complexity, Boolean network construction to implement logic relationships, and formal concept analysis (FCA) for the formation of minimal, but complete rule sets from the data.

**Results:**

First, we extracted literature information to develop an interaction network containing 18 genes representing ECM formation and destruction. Subsequently, we constructed an asynchronous Boolean network with biologically plausible time intervals for mRNA and protein production, secretion, and inactivation. Experimental gene expression data was obtained from SFB stimulated by TGFβ1 or by TNFα and discretised thereafter. The Boolean functions of the initial network were improved iteratively by the comparison of the simulation runs to the experimental data and by exploitation of expert knowledge. This resulted in adapted networks for both cytokine stimulation conditions.

The simulations were further analysed by the attribute exploration algorithm of FCA, integrating the observed time series in a fine-tuned and automated manner. The resulting temporal rules yielded new contributions to controversially discussed aspects of fibroblast biology (e.g., considerable expression of *TNF *and *MMP9 *by fibroblasts stimulation) and corroborated previously known facts (e.g., co-expression of collagens and MMPs after TNFα stimulation), but also revealed some discrepancies to literature knowledge (e.g., *MMP1 *expression in the absence of FOS).

**Conclusion:**

The newly developed method successfully and iteratively integrated expert knowledge at different steps, resulting in a promising solution for the in-depth understanding of regulatory pathways in disease dynamics. The knowledge base containing all the temporal rules may be queried to predict the functional consequences of observed or hypothetical gene expression disturbances. Furthermore, new hypotheses about gene relations were derived which await further experimental validation.

## Background

Rheumatoid arthritis (RA) is characterised by chronic inflammation and destruction of multiple joints perpetuated by the synovial membrane (SM). A major component of the inflamed SM (also called pannus tissue) are activated, semi-transformed synovial fibroblasts (SFB) [1-7]. In normal joints, SFB show a balanced expression of proteins, regulating the formation and degradation of the extracellular matrix (ECM). In RA, however, SFB are known for predominant expression and secretion of pro-inflammatory cytokines and tissue-degrading enzymes [4,5], thus maintaining joint inflammation, degradation of ECM components, and invasion of cartilage and bone. In addition, fibrosis of the affected joints is also driven by SFB, which express enhanced amounts of ECM components such as collagens [8].

Central transcription factors (TFs) involved as key players in RA pathogenesis [9,10] and in the activation of SFB in RA patients are AP-1, NF-κB, Ets-1, and SMADs [9,11-13]. These TFs show binding activity for their cognate recognition sites in the promoters of inflammation-related cytokines (e.g., TNFα, IL1β, IL6; [1]) and matrix-degrading target genes [9,10,14-17], e.g., collagenase (MMP-1 [1]) and stromelysin1 (MMP-3 [16]). The latter two show high expression levels in RA [18-20] and contributes to tissue degradation [21] by destruction of ECM components, including aggrecan or collagen types IV, X, and XI [22,23].

Due to the rapid accumulation of data about biological processes and molecular interrelationships, there is an increasing demand for approaches to analyse the underlying regulatory networks. For instance, a recent analysis of the mRNA expression profiles in synovial tissue from RA patients revealed inter-individual and gene-specific variances [24]. The underlying mechanisms for such complex behaviour are not understood so far. Mathematical and computational models may assist biologists in further research activities by generating predictions and hypotheses that can be experimentally tested. Network models, generated on the basis of extracted information and/or experimental data, will considerably facilitate the analysis of interactions among different key molecules and provide new insights into complex biological pathways and interactions (for an overview of methods see [25] and [26]). This is of particular importance in the context of rheumatic diseases and cartilage/bone metabolism, since the development of new and/or adapted molecular therapies depends on the understanding of superordinate pathway interrelationships in the pro-inflammatory micro-environment of the joint [4].

Therefore, we developed a method for simulating the temporal behaviour of regulatory and signalling networks. It was used to create a gene regulatory network emulating ECM formation and destruction, based on literature information about SFB on the one hand and on experimental data on the other, which we obtained from TGFβ1 or TNFα stimulated SFB.

The final simulations were analysed by the attribute exploration algorithm of formal concept analysis (FCA), a mathematical discipline that has multiple applications in various domains such as knowledge representation, data mining, semantic web, or software engineering. First FCA approaches related to the analysis of gene expression data have been published (for example [27,28]). Using our method, the simulation results and the observed time series were further integrated in a fine-tuned and automated manner resulting in sets of rules that determine system dynamics.

Corresponding to the discrete approach of FCA, we applied Boolean network architecture for modelling. Boolean network models, first proposed by Kauffman *et al*. [29], use binary variables that define the expression of a gene i, represented by a network node, as on or off (active or inactive, i.e., expression signal present or absent). In biology, this concept is reflected by distinct expression thresholds which must be exceeded by each individual gene to initiate their cellular effects, including disease initiation and progression [30]. Boolean networks are dynamic models and thus, they require time-series data as input ("reverse engineering") and generate such data as output ("simulation"). They can be represented as directed graphs, with the nodes labelled by Boolean functions. Boolean networks are widely used in molecular biology for logical analysis and simulation of medium or large scale networks [31,32]. For example, Kervizic *et al*. developed a method for the cholesterol regulatory pathway in 33 species which eliminates spurious cycles in a synchronous Boolean network model [33]. Sets of Boolean rules are also applied as so-called knowledge bases in decision support or expert systems.

For our analysis we used a collection of 18 genes, which can be classified into five functional groups, sufficient to create a self-contained regulatory network of ECM maintenance: (1) structural proteins which are the target molecules (i.e., the collagen I-forming subunits *COL1A1 *and *COL1A2*); (2) enzymes degrading them (i.e., the matrix metalloproteinases *MMP1*, -*3*, -*9*, and -*13*); (3) molecules that inhibit these proteases (tissue inhibitor of metalloproteinases *TIMP1*); (4) TFs (i.e., *ETS1*, *FOS*, *JUN*, *JUNB*, *JUND*, *NKFB1*) and modulators acting as TFs (i.e., *SMAD3*, *SMAD4*, *SMAD7*) which are regulated by (5) the external signalling molecules TNFα (*TNF*) and TGFβ1 (*TGFB1*). These genes (Table 1) are known to be expressed by SFB, except for *TNF *and *MMP9*, for which the expression is still under question (see below).

**Table 1:** List of genes used in this analysis.

**Name**	**Protein name**	**Protein class**	**Main function**
*COL1A1*	Type I collagen alpha 1 chain	Structural component	Cartilage and connective tissue collagen
*COL1A2*	Type I collagen alpha 2 chain	Structural component	Cartilage and connective tissue collagen
*MMP1*	Matrix metalloprotease 1 (interstitial collagenase)	Protease	Cleavage of collagen I, II, and III
*MMP3*	Matrix metalloprotease 3 (stromelysin 1)	Protease	ECM component degradation
*MMP9*	Matrix metalloprotease 9 (gelatinase B)	Protease	Cleavage of collagen IV and V
*MMP13*	Matrix metalloprotease 13 (collagenase 3)	Protease	Cleavage of collagen I and III
*TIMP1*	Tissue inhibitor of metalloprotease 1 (collagenase inhibitor)	Protease inhibitor	Inhibits matrix metalloproteases, and others
*ETS1*	v-ets erythroblastosis virus E26 oncogene homolog 1	Transcription factor	Erythroblast and fibroblast transformation
*FOS*	v-fos FBJ murine osteosarcoma viral oncogene homolog	Component of AP-1	Regulator of proliferation, differentiation, and transformation
*JUN*	v-jun avian sarcoma virus 17 oncogene homolog	Component of AP-1	Regulator of proliferation, differentiation, and transformation
*JUNB*	jun B proto-oncogene	Component of AP-1	Regulator of proliferation, differentiation, and transformation
*JUND*	jun D proto-oncogene	Component of AP-1	Regulator of proliferation, differentiation, and transformation
*NKFB1*	Subunit 1 of nuclear factor kappa-B	Component of NF-κB	Involved in many biological processes
*SMAD3*	SMA- and MAD-related protein 3	Transcriptional modulator	Mediator of signal transduction by TGFβ1 (RSMAD)
*SMAD4*	SMA- and MAD-related protein 4	Transcriptional modulator	Mediator of signal transduction by TGFβ1 (CoSMAD)
*SMAD7*	SMA- and MAD-related protein 7	Transcriptional modulator	Antagonist of signalling by TGFβ1 (ISMAD)
*TNF*	Tumour necrosis factor alpha	Cytokine	Proinflammatory role
*TGFB1*	Transforming growth factor beta 1	Growth factor	Involved in proliferation and differentiation

## Results and discussion

### Creating a regulatory network by literature extraction

The available literature was screened for genes and proteins involved in ECM maintenance and expressed in the lining layer SFB of the SM. In order to derive a regulatory network, we comprehensively collected literature knowledge related to the formation and degradation of ECM in human fibroblasts and analysed it manually. We chose collagen type I, which is formed by the *COL1A1 *and *COL1A2 *gene products, as a connective tissue representative, several MMPs as ECM-degrading enzymes, their inhibitors, and TFs regulating them. Finally, we selected 18 genes (Table 1) and the literature was screened again for gene regulatory relations and interactions connecting them (see additional file 1 for a complete list). Some contradictory literature findings were resolved manually (see section Boolean functions adapted to the data).

The resulting regulatory network is almost closed and represents the most important ECM network functions. Here, we imply that the receptors for the external signalling molecules are always available and functional in SFB. Note, that TGFβ1 (*TGFB1*) and TNFα (*TNF*) are the only entities playing a dual role as both external signal molecules and target genes because of their introduction into the simulation as starting effectors.

It turned out that the knowledge about gene regulatory events is limited and that, to the best of our knowledge, the regulation of SMAD and SMAD expression has not been fully characterised so far. The SMAD gene products seem to be available in sufficient amounts and we were unable to find reports about their regulated expression. In addition, not all influences of TGFβ1 and TNFα on gene expression could be described as direct effects of transcription factors at the mRNA level because the important SMAD family members act as regulators on the protein-protein interaction level. All influences were included in the network at this point to avoid premature loss of information.

Although many TFs such as AP-1 are also regulated at the protein level (e.g., by phosphorylation), those effects can be reflected simplistically by regulatory processes at the transcriptional level. However, activating SMADs as SMAD3 and SMAD4 are also regulated by inhibitory members of the SMAD family (SMAD6 and SMAD7), which may counteract transcriptional activation and add an extra level of complexity [34]. Therefore, SMAD7 was introduced into the network as a TGFβ1-dependent repressor of SMAD-dependent transcription.

In the case of SMAD3, we decided to subsume its influence under the SMAD4 effects because both are described to have nearly identical effects and act in concert. Moreover, we could not find well-defined information about SMAD3 regulation. Hence, we added an inducing influence of SMAD4 on *MMP13 *(at present only known for SMAD3) for keeping all the SMAD effects in the network.

**Figure 1:**
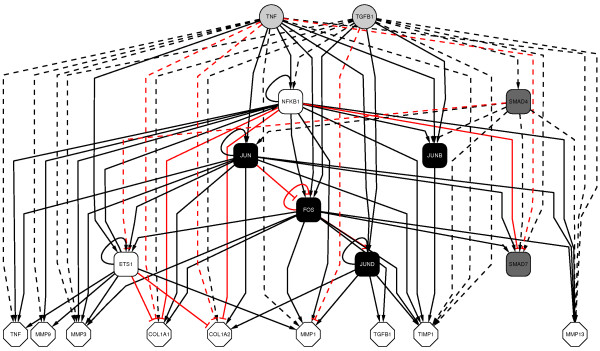
**Overview of the ECM network in hierarchical order**. Regulatory effects *via *TF s are shown as continuous lines, others as indirect effects as dashed lines. Inhibition is marked by a red T-arrow, induction is illustrated by black arrows. The external signals TGFβ1 and TNFα are shown as light grey circles, the internal SMAD signalling molecules as dark grey squares, TFs are depicted as black (AP-1 components) or white squares, and the target genes are shown as white octagons. This picture was generated using Cytoscape 2.6.0.

The subunits of the homo- or heterodimer TF AP-1, i.e., Jun, JunB, JunD, and Fos (*JUN*, *JUNB*, *JUND*, *FOS*), determine its different regulatory activities (for AP-1 components see [35] and references therein). Therefore, we decided to disassemble the transcriptional active entity AP-1 into its subunits. In contrast, for the dimeric TF NF-κB, which composed of the gene products of *NFKB1*, *NFKB2*, *RELA*, *RELB*, and/or *REL *[36], we selected *NFKB1 *as the representative gene with respect to our signalling framework. All the genes and their interrelations were transferred into the program Cytoscape [37] to visualise our network containing 19 nodes and 79 edges, respectively, as shown in Figure 1. Detailed network examination is available through the network description files (additional files 2 and 3), also providing external links to GenBank, Uniprot, and PubMed for all edges and nodes.

Available tools for automatic text mining decide schematically, e.g., by pre-built rules like co-occurrence of gene names and interaction verbs or pattern matching, whereas a human expert is able to integrate unanticipated types of information and to decide whether the paper confirms the investigated situation. However, we used the tools Bibliosphere [38] and Pathway Studio [39] in order to verify completeness and consistency of the assembled network (data not shown).

### Boolean functions

Due to its capability for displaying dynamic dependencies between individual parameters, a Boolean network is more specific than the graphical network in Figure 1, which summarises isolated literature facts. In order to decide about the connectives OR/AND, which represent causally determined relations between different genes, cellular signalling processes were also considered.

In the case of a known transcriptional activation of any gene by the stimuli TNFα or TGFβ1 *via *a specific TF, this activation was represented in the network using the term GENE.out = STIMULUS AND TF. Without such evidence, these influences were connected by GENE.out = STIMULUS OR TF. Since it is well known that the so-called SMAD pathway is activated by TGFβ1 but not influenced by TNFα [40], we used the *AND *connection for SMAD3/4 and TGFβ1, even if there was no explicit literature evidence for an impact of TGFβ1 onto the respective gene.

Another example for setting up the functions is the integration of: (i) the known auto-regulatory transcriptional activation of *JUN *by TNFα *via *JUN, and (ii) the activation of *JUN via *SMAD4 (TNFα – independent) into the single Boolean function 5 (compare Table 2 with Tables 3 and 4): JUN.out = (TNF AND JUN) OR (TGFB1 AND SMAD4). Based on the illustrated principles, the Boolean functions characterising formation and remodelling of the ECM were generated (Table 2).

**Table 2:** Boolean functions based on literature information.

1	COL1A1.out	= (TGFB1 OR FOS OR JUN) AND NOT (TNF AND (NFKB1 OR ETS1))
2	COL1A2.out	= ((TGFB1 AND JUND) OR JUN) AND NOT (TNF AND (NFKB1 OR ETS1))

3	ETS1.out	= ((TNF AND (ETS1 OR JUN)) OR FOS) AND NOT SMAD4

4	FOS.out	= (TGFB1 OR (TNF AND NFKB1)) AND NOT (JUN AND FOS)

5	JUN.out	= (TNF AND JUN) OR SMAD4

6	JUNB.out	= (TGFB1 AND NFKB1 AND SMAD4) OR (TNF AND NFKB1)

7	JUND.out	= (TGFB1 OR TNF) AND JUND AND NOT FOS

8	MMP1.out	= ((TNF AND (ETS1 OR NFKB1)) OR FOS OR JUND) AND NOT TGFB1

9	MMP3.out	= (TNF AND (ETS1 OR JUN OR NFKB1)) OR FOS OR TGFB1

10	MMP9.out	= TNF AND (ETS1 OR NFKB1)

11	MMP13.out	= (TNF AND (JUN OR NFKB1)) OR FOS OR (TGFB1 AND SMAD4)

12	NFKB1.out	= (TNF AND (ETS1 OR NFKB1)) OR TGFB1

13	SMAD4.out	= TGFB1

14	SMAD7.out	= ((TGFB1 AND SMAD4) OR FOS OR JUN) AND NOT (TNF AND NFKB1)

15	TIMP1.out	= (TGFB1 AND SMAD4) OR (TNF AND (JUN OR JUNB OR JUND OR NFKB1)) OR FOS

16	TGFB1.out	= FOS OR JUND

17	TNF.out	= TNF AND (ETS1 OR JUN OR NFKB1)

**Table 3:** Revised Boolean functions for the simulation of TGFβ1 stimulation.

**1**	COL1A1.out	= TGFB1 OR FOS OR JUN	*
**2**	COL1A2.out	= (TGFB1 AND JUND) OR JUN	*

**3**	ETS1.out	= FOS ***AND NOT (TGFB1 AND SMAD4 AND NOT SMAD7)***	*

4	FOS.out	= (TGFB1 OR (TNF AND NFKB1)) AND NOT (JUN AND FOS)	

5	JUN.out	= (TNF AND JUN) OR (***TGFB1 AND ***SMAD4 ***AND NOT SMAD7***)	

6	JUNB.out	= (TGFB1 AND NFKB1 AND SMAD4 ***AND NOT SMAD7***) OR (TNF AND NFKB1)	

7	JUND.out	= TGFB1 OR TNF ***OR ***(JUND AND NOT FOS)	

**8**	MMP1.out	= JUND AND NOT (TGFB1 ***AND FOS***)	*

**9**	MMP3.out	= (TNF AND ((ETS1 ***AND ***NFKB1) OR JUN)) OR FOS	

10	MMP9.out	= TNF AND ETS1 ***AND ***NFKB1	

**11**	MMP13.out	= (TNF AND (JUN OR NFKB1)) OR FOS	

**12**	NFKB1.out	= TNF ***OR ***ETS1 OR NFKB1	*

13	SMAD4.out	= TGFB1	*

**14**	SMAD7.out	= (TGFB1 AND SMAD4 ***AND NOT SMAD7***) OR FOS OR JUN	*

15	TIMP1.out	= (TGFB1 AND SMAD4 ***AND NOT SMAD7***) OR (TNF AND (JUN OR JUNB OR JUND OR NFKB1)) OR FOS	

16	TGFB1.out	= FOS OR JUND	

17	TNF.out	= TNF AND ((ETS1 ***AND ***NFKB1) OR JUN)	

**18**	Inactivation rule	***If the transcription factors ETS1, FOS, JUN or JUNB are expressed at t > 0, they will be down- regulated at t+3 and afterwards*.**	

Rows marked by an asterisk indicate differences of the functions for TGFβ1 and TNFα stimulation, changes to Table 2 are italicised and printed in bold. Function numbers in bold indicate omitted (1, 2, 3, 8, 9, 11, 12, 14) or inserted (18) terms.

**Table 4:** Revised Boolean functions for the simulation of TGFα stimulation.

**1**	COL1A1.out	= (FOS OR JUN) AND NOT (TNF AND ETS1 ***AND ***NFKB1)	*
**2**	COL1A2.out	= JUN AND NOT (TNF AND ETS1 ***AND ***NFKB1)	*

3	ETS1.out	= (TNF AND (ETS1 OR JUN)) OR FOS	*

4	FOS.out	= (TGFB1 OR (TNF AND NFKB1)) AND NOT (JUN AND FOS)	

5	JUN.out	= (TNF AND JUN) OR (***TGFB1 AND ***SMAD4 ***AND NOT SMAD7***)	

6	JUNB.out	= (TGFB1 AND NFKB1 AND SMAD4 ***AND NOT SMAD7***) OR (TNF AND NFKB1)	

7	JUND.out	= TGFB1 OR TNF ***OR ***(JUND AND NOT FOS)	

**8**	MMP1.out	= (TNF AND ETS1 ***AND ***NFKB1) OR FOS OR JUND	*

**9**	MMP3.out	= (TNF AND ((ETS1 ***AND ***NFKB1) OR JUN)) OR FOS	

10	MMP9.out	= TNF AND ETS1 ***AND ***NFKB1	

**11**	MMP13.out	= (TNF AND (JUN OR NFKB1)) OR FOS	

**12**	NFKB1.out	= TNF AND (ETS1 OR NFKB1)	*

**13**	SMAD4.out	= ***TRUE***	*

**14**	SMAD7.out	= (FOS OR JUN) AND NOT (TNF AND NFKB1)	*

15	TIMP1.out	= (TGFB1 AND SMAD4 ***AND NOT SMAD7*) **OR (TNF AND (JUN OR JUNB OR JUND OR NFKB1)) OR FOS	

16	TGFB1.out	= FOS OR JUND	

17	TNF.out	= TNF AND ((ETS1 ***AND ***NFKB1) OR JUN)	

**18**	Inactivation rule	***If the transcription factors ETS1, FOS, JUN or JUNB are expressed at t > 0, they will be down-regulated at t+3 and afterwards***.	

Rows marked by an asterisk indicate differences of the functions for TGFβ1 and TNFα stimulation. changes to Table 2 are italicised and printed in bold. Function numbers in bold indicate omitted (1, 2, 8, 9, 11, 12, 13, 14) or inserted (18) terms.

### Gene expression time courses following TGFβ1 and TNFα stimulation

We analysed gene expression changes of SFB from patients with RA (3 patients) or OA (3 patients) following TGFβ1 and TNFα stimulation (Table 5). Due to the strong stimuli, both groups of cells reacted in an almost identical way, and we did not differentiate among them. In another study, for example, OA cells were considered to be a disease control group [41].

**Table 5:** Clinical characteristics of the patients at the time of synovectomy/sampling.

		**Rheumatoid**	**Osteoarthritis**
		**Arthritis (RA)**	**(OA)**
Patients (n)		3	3

Sample name		RA1, RA2, RA3,	OA1, OA2, OA3

Gender (female/male)		3/0	1/2

Age (years ± SEM)		62.4 ± 2.9	58.7 ± 2.0

Disease duration (years ± SEM)		11.5 ± 0.5	4.4 ± 0.6

Rheumatic factor (positive/negative)		3/0	0/3

ESR^1 ^(mm/h ± SEM)		26.7 ± 6.2	20.0 ± 4.0

CRP^2 ^(mg/l ± SEM)		38.1 ± 7.2	13.2 ± 2.9

ARA^3 ^– Criteria for RA (n ± SEM)		6.0 ± 1.1	0.3 ± 0.1

Concomitant	MTX^4 ^(n)	1	0
	
medication	NSAIDs^5 ^(n)	3	3

^1 ^Erythrocyte sedimentation rate

^2 ^C-reactive protein, normal range: < 5 mg/l

^3 ^American Rheumatism Association (now: American College of Rheumatology)

^4 ^Methotrexate

^5 ^Non steroidal anti-inflammatory drugs

**Figure 2:**
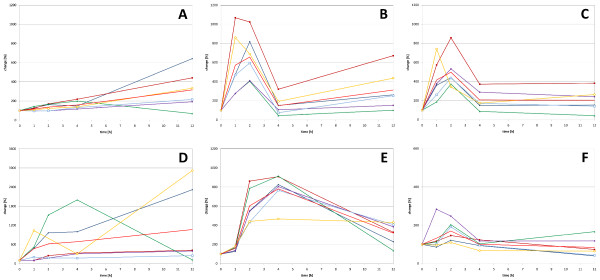
**Gene expression time courses following TGFβ1 or TNFα treatment**. *COL1A1 *(A), *JUNB *(B), and *SMAD7 *(C) gene expression in response to TGFβ1 treatment (upper row); TNFα response (lower row) of *NFKB1 *(D), *MMP1 *(E), and *SMAD7 *(F). The average time course is shown as light red curve without symbols, the data for individual samples are depicted in other colours (OA1: blue, filled symbol; OA2: red, filled symbol; OA3: green, filled symbol; RA1: purple, filled symbol; RA2: blue, open symbol; RA3: yellow, open symbol). The time courses and the values calculated from the microarray experiments for all analysed genes are included in additional file 4.

Following pre-processing of the microarray data gained from U133 Plus 2.0 arrays, we extracted the data for probe sets related to our genes of interest (see Methods). The data are available in the GEO database (GSE13837 at [42]). For the following analyses we excluded values which exceeded the reliability threshold of p ≤ 0.05 for any patient at any time point (0, 1, 2, 4, 6, and 12 hours). In Figure 2, some selected examples for the influence of TGFβ1 and on gene expression are presented. The time courses of *COL1A1 *and *JUNB *expression are shown to illustrate the TGFβ1 response in SFB, and the TNFα response is illustrated by *NFKB1 *and *MMP1 *expression. *SMAD7 *expression data are also included for both treatments. The data and the respective diagrams for all genes and both treatments can be found in additional file 4.

For comparative purposes, we also analysed public data from the GEO database, first, TGFβ1 treated murine embryonic fibroblasts (GSE1742) and second, TNFα stimulation of endothelial cells (HeLa, GSE2624). Following prolonged TGFβ1 treatment in murine cells, *COL1A2*, *JUN*, and *TIMP1 *gene expression increased, whereas *FOS *decreased. In contrast, *FOS*, *JUN*, and *JUNB *expression in HeLa cells rapidly increased following TNFα stimulation. Unfortunately, no data about the protease genes were available in this dataset (additional file 5). Even though cell type, experimental design and duration of treatment differ from our experiments, they reflect the two general trends: a positive effect on ECM formation by TGFβ1 and a degradative influence on ECM by TNFα (mediated at least in part by FOS and JUN), which is consistent with our data. However, the evaluation of the complete data sets revealed discrepancies between the expected expression profile of individual genes and their time courses following stimulation in the experiment.

### Data discretisation

We developed a data discretisation method which appropriately captures biologically relevant differences in gene expression levels. The individual time profiles for each gene were separately discretised to the values 0or 1 by k-means clustering [43], a method which is often applied for gene expression time series. No improvements were observed when applying Ward's hierarchical clustering [44] or single linkage clustering as proposed in [45] (data not shown). We introduced several supplementary criteria (see Methods), e.g., the values of a time series were all discretised to the constant value 0 or 1, if the differences of all log2 values (fold-changes) were less than 1 [46]. For the discretised data see additional file 6.

### Boolean functions adapted to the data

Simulations were generated using an asynchronous update scheme, assuming time intervals – approximately equal to 1 h time steps – as follows: transcription 1 (NFKB1: 2), translation: 1, RNA lifespan: 1, and protein lifespan: 2. The Boolean functions generated the transcriptional states according to the functional influence of proteins (stimuli or TF); translation and mRNA/protein degradation were computed from this output state according to the predefined intervals (see Principles of simulation in Methods section).

As starting conditions of the simulations we chose the discretised initial states derived from our experimental data. An additional initial state was introduced in which solely the transcription factors were set to on, which enables the model system to respond to the external stimulators TNFα or TGFβ1 immediately. The simulations were performed over twelve time steps; however, we did not aim at an exact correspondence to the experimental observation time of twelve hours, but tried to adjust the simulated time courses to qualitative features such as early, intermediate or late up-regulation. Improving the Boolean functions accordingly, the initially applied literature-based information was completed by: (i) valid and specific experimental information; (ii) knowledge and experience of biological experts; and (iii) in some cases, a more focused and precise literature query (Tables 3 and 4). For a comparison of the discretised observed time series and the final simulations see the additional files 6 and 7. We developed several biologically interesting and plausible data-independent hypotheses, for example, we modelled the regulation of SMAD3/SMAD4 effects by a protein-protein interaction with SMAD7.

The resulting optimised Boolean network with the revised Boolean functions (Tables 3 and 4) represents an enhanced ECM model, roughly matching the given biological conditions and extensively exceeding the present possibilities of automatic methods such as text mining, symbolic computation or machine learning. Considering the additional information available, we accepted these biologically reasonable changes:

1. In the case of TNFα (or TGFβ1) stimulation, the production and secretion of TGFβ1 (or TGFα) by SFB should not contradict the influence of the abundant stimulating protein TNFα (or TNFβ1). In these cases (e.g., for the COL1A1.out function in Table 4, and for the MMP1.out function in Table 3) we removed TGFB1 (TNF) AND (...) from the Boolean function term. This adjustment did not always change the simulation result, since, for example, TNF was always off following TGFβ1 stimulation (numbers of the Boolean functions (BF) affected: 1, 2, 3, 8, 9, 11, 12, and 14).

2. Down-regulation of gene expression is an essential biological principle. For that reason we had to introduce a time-limited inactivation mechanism which could not be derived from the literature because information regarding down-regulatory mechanisms is very restricted. Moreover, complex and variable mechanisms were hard to model, e.g., *JUN *down-regulation which is driven by: (i) inactivation of the TF protein itself; (ii) a general shift in the composition of the TF AP-1, resulting in a reduced amount of TF enhancing *JUN *transcription; and (iii) binding/inactivation of JUN by other proteins. Therefore, a time-limited mRNA inactivation was introduced for *JUN*, *JUNB*, *FOS*, and *ETS1*. Accordingly, an inactivating rule was created: if these TFs are expressed at t> 0, they will be set to off at t+3 and afterwards (no. of BF affected: 18). In addition, at that step we included an inhibition of TGFB1/SMAD4 signalling-based target gene expression by integrating a SMAD4-inhibiting signal (i.e., SMAD7, included as AND NOT SMAD7) guaranteeing the subsequent inactivation of TGFβ1-related gene expression (BF affected: 3, 5, 6, 14, and 15). *JUND *is constitutively expressed at an intermediate level, which is consistent with GEO (GSE1742 and GSE2624) and our own data, as well as with the literature [47]. For *NFKB1 *transcription, an inhibitory effect was not implemented, since the activity of NF-κB at the protein level is controlled by interaction with several IKB proteins [48] which were not included in our ECM network model.

3. *SMAD4 *induction is not dependent on TGFβ1 stimulation, because it is constitutively expressed (i.e., always TRUE, BF affected: 13). However, without TGFβ1-mediated phosphorylation, SMAD4 is not activated at the protein level and shows no transcriptional activity, even though constitutively expressed. Therefore, we amended the term SMAD4 to TGF AND SMAD4 in order to represent the necessity of TGFβ1 for SMAD4 activation (BF affected: 3, 5).

4. We considered the relation ETS1 AND NFKB1 for the target genes [49] instead of assuming alternative pathways by ETS1 OR NFKB1 because regulation by NF-κB seems to be dependent on ETS1, and the MMPs, for example, require both factors [50] (BF for TGF stimulation affected: 9, 10, and 17, Table 3; BF for TNF stimulation affected: 2, 8, 9, 10, and 17, Table 4).

5. Since the inhibition of *JUND *expression by FOS is only observed in the case of a concomitant *JUND*-based positive feedback, the inhibitory effect of FOS has been restricted to this case [51] (BF affected: 7).

6. Since a TF should not necessarily be required for its own expression (positive feedback), in the case of *JUND *(and also *NFKB1*) the AND connection was changed to OR. The revision of this function prevents the absence of *JUND *expression following TGFβ1 stimulation (BF affected: 7, 12).

7. Concerning the regulation of *MMP1 *expression by FOS, there were contradictory findings in the literature [52,53]. We decided for an inhibitory influence of FOS following TGFβ1 stimulation, because otherwise *MMP1 *would have been permanently down-regulated by TGFβ1 during the simulation (BF affected: 8, Table 3).

8. The Boolean function MMP3.out = (...) OR TGFB1 was in obvious contradiction to the data of the present study, thus, the term OR TGFB1 was deleted. The same was done for the *MMP13 *function (BF affected: 9, 11).

9. In the case of *NFKB1*, the absence of TNFα stimulation had no decisive influence (*NFKB1 *was not always off). For that reason, we changed NFKB1.out = TNF AND (ETS1 OR NFKB1) to NFKB1.out = TNF OR ETS1 OR NFKB1 (BF affected: 12, Table 3).

10. However, concerning the expression of *TNF *itself, the necessity of a positive feedback could explain its complete absence following TGFβ1 stimulation. On the other hand, *TNF *was expressed at some time points following commonly assumed that fibroblasts do not express TNF (BF not changed: 17).

In summary, we adjusted the set of BF obtained by adaptation to the gene expression data measured under two experimental conditions (TNFα and TGFβ1 stimulation), in order to create an appropriate set of BF representing the existing knowledge about naturally occurring interrelationships as accurately as possible.

### Computing temporal rules by attribute exploration

For each stimulus, the observed and the (final) simulated time series were translated and merged into a single formal context, the basic tabular data structures of FCA (see section Creation of a temporal rule knowledge base in the Methods section). States are defined by the value on or off for each gene, and transitions were computed by linking an occurring input state to an arbitrary future (output) state of the simulation or observation. The set of all these transitions (formal context, compare Table 6) was analysed by the automatic, noninteractive version of the attribute exploration algorithm, which computes a minimal and complete knowledge base (stem base), from which all implications of a given formal context can be derived [54].

The implications of the stem base are temporal rules expressing hypotheses about attributes of states (e.g., co-regulation or mutual exclusion of gene expression) or system dynamics, which are supported by pre-existing knowledge and by the analysed data. Since an implication holds for the transitions between all temporally related states, a rule such as GENE1.in.on → GENE2.out.on means: if gene1 is expressed, gene2 always will be up-regulated in the future, at all subsequent observation time points, and simulation steps. Due to this semantics, the implications neither depend on the correspondence of a simulation time step to a specific observation interval, nor on prior knowledge about time periods of direct or indirect transcriptional effects. Within the large knowledge bases for TNFα (8,785 rules) and TGFβ1 (2,713 rules) stimulation, the most frequent and simple temporal rules were considered and analysed for dependencies between stimuli, induced TFs, and their target genes.

**Table 6:** Example for the observed transition context *K*_*obs*_. The rows represent the transitions from state *n *to the state (*n+1*) of sample OA1 following TNFα stimulation.

**Attribute →**												
	**FOS.in**	**JUN.in**	**JUNB.in**	**JUND.in**	**MMP3.in**	**...**	**FOS.out**	**JUN.out**	**JUNB.out**	**JUND.out**	**MMP3.out**	**...**
**Transition ↓**												
**(*s*_0_^*in*^,*s*_1_^*out*^)**	on	off	off	on	off	...	on	on	on	on	on	...
**(*s*_0_^*in*^,*s*_2_^*out*^)**	on	off	off	on	off	...	off	on	on	on	on	...
**(*s*_0_^*in*^,*s*_4_^*out*^)**	on	off	off	on	off	...	off	off	off	on	on	...
**(*s*_0_^*in*^,*s*_12_^*out*^)**	on	off	off	on	off	...	on	off	off	on	on	...
**(*s*_1_^*in*^,*s*_2_^*out*^)**	on	on	on	on	on	...	off	on	on	on	on	...
**...**	...	...	...	...	...	...	...	...	...	...	...	...

### Results of the attribute exploration

#### Stimulation with TNFα

Regarding stimulation with TNFα, a coordinated down-regulation with of the two TF SMAD7 (inhibitor of TGFβ1/SMAD4 signalling) and ETS1 emerges, as indicated by the rules 33, 114, 135, 144, 157, and 186 (see additional file 8). For example, rule 186:

<90> COL1A1.out.off, EST1.out.off → SMAD7.out.off

has the meaning: in all simulated and observed states characterised by the absence of COL1A1 and ETS1, SMAD7 is also off. <90> stands for the support of the rule, i.e., the number of transitions (90 out of 294) that actually have the attributes of the premise. Rules 114, 135, 144, 157, and 186 indicate: if the TNFα-dependent genes are not induced (ETS1 as mediator), then simultaneously the expression of TGFβ1-dependent genes is enabled (SMAD7 is off). This suggests that TNFα and TGFβ1 may act as antagonists in SFB, as described in [55,56].

The expression of *NFKB1*, which is also induced by TNFα, proceeds conversely to that of *ETS1 *and *SMAD7 *(rules 34, 45, 70, 71, 134, 144, 154, 157, and 173) reflecting the different targets of NF-κB and SMAD7. The antagonistic pattern of *NFKB1 *and *SMAD7 *appears indirectly in rule 33, where the two genes show up in the premise of a rule with high support:

<150> (...) NFKB1.out.on, SMAD7.out.off → EST1.out.off

Regarding this rule, it is interesting that ETS1 always acts in the same direction as NF-κB, according to the network derived from the literature (Figure 1). In the adapted network (Table 4), we assumed a necessary cooperation (i.e., an AND connective) for the positive regulation of *ETS1*, *MMP1*, *MMP3*, *MMP9*, and *TNF*, as well as for the inhibition of *COL1A1 *and *COL1A2*. Thus, rule 33 further suggests that the coordinated action of NF-κB and ETS1 is turned off in states which are characterised by supplementary conditions as SMAD7.out.off.

The generated rules adequately reflect the major influence of the TF AP-1 in the system: the expression of prominent targets, such as *COL1A1*, *MMP1*, and *MMP3*, depends on JUN (rules 211 and 258) and/or FOS (rule 204), with JUN as the key player. These rules connect input and output states and thus their semantics is directly related to dynamics, as seen in rule 211:

<87> TGFB1.in.on, TIMP1.in.on, ETS1.in.on, JUN.in.on → MMP1.out.on

making this strong statement: if *ETS1 *and *JUN *are on, *MMP1 *will always be up-regulated in the future (at least within the time frame of 12 hours).

Sometimes, *MMP1 *is expressed simultaneously or before *ETS1 *and *JUN*. In the simulation, *MMP1 *was always *on *in the output state and from time point 2 h in the data. An exception can be found for the experimental results from OA sample OA3 (Table 5), where *MMP1 *is off after 12 h. This is the reason for the computation of the auxiliary conditions TGFB1.in.on and TIMP1.in.onin rule 211.

Concerning the regulation of target genes, the expression of *MMP1*, *MMP3*, and *MMP13 *is co-regulated (rules 35, 63, 82, 86, and 176), while *MMP9 *is expressed independently (rules 24 and 35). There is a contradiction between the simulation and the data: in the observed experimental time series, *MMP13 *is always off, whereas the Boolean network predicts an up-regulation similar to *MMP1 *and *MMP3*. This unexpected absence of predicted *MMP13 *expression may be an indication for a more complex regulation of *MMP13 *transcription, exceeding the already known regulatory interrelations. Therefore, the *MMP13 *promoter and further enhancer/repressor sequences should be targeted for a more pronounced structural and functional analysis. For *MMP9*, the simulation and the experimental data are in good agreement: the gene is off in most, but not all states. However, since the expression of *MMP9 *by (S)FB is discussed controversially in the literature (see [57] and [58]*vs*. [59]), the calculated expression of *MMP9 *by fibroblasts – at least at a limited number of time points – supports the majority of studies, reporting detectable *MMP9 *mRNA amounts in (S)FB.

Several rules unanimously indicate the co-expression of the ECM-forming genes *COL1A1 *and *COL1A2 *(rules 87, 88, and 95), but contradictory rules occur concerning their expression profile in comparison to the MMPs. *COL1A1 *and *COL1A2 *seem to be co-expressed with *MMP1 *(rules 90 and 176), for *COL1A2*, however, a certain co-expression with *MMP9 *is calculated as well (rules 76 and 77), which conflicts with the opposing expression of *MMP1 *and *MMP9 *(see above). Therefore, the expression of collagens does often, but not necessarily always correlate with the expression of MMPs. This reflects the imbalance between MMP-dependent destruction and collagen-driven regeneration/fibrosis of ECM in the joints in inflammatory RA.

The calculated knowledge base also contains a further unexpected correlation. According to rule 166:

<94> FOS.in.off, TIMP1.in.on, SMAD7.out.off → TGFB1.in.on, MMP1.out.on, TGFB1.out.on

and rule 188, the expression of *MMP1 *may also be induced in the absence of FOS (e.g., by JUN-containing AP-1 complexes), indicating that the regulation of *MMP1 *does not predominantly depend on FOS as proposed in the literature [17,60]. This result may point to the influence of other TFs, e.g., NF-κB, ETS1, or AP-1 complexes containing JUN, which may indeed be able to induce target gene expression in the absence of FOS.

#### Stimulation with TGFβ1

For the stimulation with TGFβ1, we had a total number of 341 transitions. The SMADs play a major role for the expression of TGFβ1-dependent target genes, as reflected by various classes of rules containing SMAD4 and/or SMAD7 (see additional file 8). For example, SMAD4 can be involved in the expression of *COL1A1*, see rule 15 (and also rules 21, 26, and 30):

<239> ETS1.out.off → SMAD4.in.on, COL1A1.out.on, SMAD4.out.on

This also suggests an antagonistic behaviour of ETS1 and SMAD4: if ETS1 was off, then SMAD4 was on, as well as in all previous states. Rules 52 and 57 suggest a dependency of MMP1 on SMAD4. However, this seems to be one amongst many other influences (or could be a non-influencing coincidence), since SMAD4 was permanently on during simulation and experimental stimulation with TGFβ1 (exception: sample RA3 at time point 2 h).

The expression of *MMP9 *is neither induced by SMAD4 (rules 7, 24, and 41) nor by any other TF, indicating that *MMP9 *is not influenced by TGFβ1. The fact that TGFβ1 obviously does not induce *MMP9 *(but other MMPs) agrees with findings reported previously [59] and represents a clear contrast to the MMP expression profiles following TNFα stimulation.

A further case of an antagonistic expression pattern was calculated for MMPs and *COL1A1 *(rules 21, 30, 36, 41, 54, and 60), for example, in rule 54:

<170> SMAD4.in.on, MMP3.out.off, MMP9.out.off, MMP13.out.off, ... → COL1A1.out.on

Antagonistic expression profiles also can be observed for *SMAD4 *and other TFs, e.g., *JUN *and *JUNB *(rules 12, 39) or *ETS1 *(rule 15, see above). The variety of TF combinations found, even following the same stimulus, exceeds the possibilities of conventional TF studies because stimulation experiments are generally restricted to a selected set of readout parameters (e.g., the expression of single TFs or target genes) which are not able to reflect the multiplicity of different effects in the cell.

Following stimulation with TGFβ1, interestingly *COL1A2 *appears to be constitutively expressed since its status is always calculated as on (rule 1). Therefore, for the formation of collagen I, which contains COL1A1 and COL1A2 chains, *COL1A1 *expression seems to be the critical switch.

#### TGFβ1 versus TNFα effects

The calculated results impressively illustrate that TGFβ1 and TNFα stimulation are mediated *via *separate signal transduction pathways, leading to the expression and activation of different TFs. In general, *ETS1 *and *NFKB1 *are induced predominantly by TNFα, whereas SMAD expression depends on TGFβ1 (represented by differential expression profiles of *ETS1 *and *SMAD4*). *JUN *and *FOS*, however, strikingly respond to both stimuli. This defined pattern results in the expression of target genes with opposing roles. TGFβ1 positively regulates the enhanced formation of ECM components, whereas TNFα is strongly involved in the expression of ECM-degrading enzymes. This was the main reason for a discriminative revision of the BF for TNFα and TGFβ1 (Tables 3 and 4). Six BF were found to be differently adjusted (BF 1, 2, 3, 8, 12, and 14), which concern either the key players for ECM destruction (MMP1; BF 8), ECM formation (COL1A1 and COL1A2; BF 1 and 2) or important regulatory genes (ETS1, NFKB1, SMAD7). This may indicate that the differential effects on ECM induced by TNFα or TGFβ1 are mainly mediated *via *ETS1 (BF3), NFKB1 (BF 12, especially in the TNFα pathway), or SMAD7 (BF 14, especially in the TGFβ1 pathway) identifying ETS1- and NFKB1-associated pathways as the major TNFα-induced pro-inflammatory/pro-destructive signalling modules in rheumatic diseases, whereas TGFβ1-driven and SMAD7-related signalling appear to be prominently involved in fibrosis.

### Querying the knowledge base

The minimal rule set gave many new insights, and further queries can be addressed by accessing the TNFα and TGFβ1 knowledge bases in one of two ways: (i) the Excel file containing the transition rules for structured searches within the rule sets (see additional file 8 containing the top 500 transition rules, additional files 9 and 10 for complete lists); and (ii) the stem base in PROLOG format for queries concerning logically implied rules (additional files 11 and 12).

## Conclusion

The analyses in the present study were based on literature data valid for healthy human SFB. These findings were fine-tuned and adapted to gene expression time course data triggered by TGFβ1 and TNFα in SFB from RA and OA patients. Both the assembly of previous knowledge and the adaptation of the Boolean functions gave detailed insight into disease-related regulatory processes. To the best of our knowledge, this is the first dynamical model of ECM formation and degradation by human SFB.

One of the strengths of the FCA method applied here is its ability to give a complete, but minimal representation of observed or simulated data. This complete overview of temporal rules enabled us to find new relationships. The most unexpected result is the expression of *TNF *at some time points following TNFα stimulation, whereas it is commonly assumed that SFB do not express *TNF *[61-64]. Similarly, our experimental data as well as our simulation results support *MMP9 *expression in SFB thus corroborating the majority of the literature regarding expression of this protease [57,58]. Here, it is important to note that a contamination of our SFB population with macrophages (potentially contributing to MMP9 production) can be excluded due to the SFB isolation protocol, resulting in a pure SFB population [65]. We also found that *MMP1 *was induced in the absence of FOS after TNFα stimulation, whereas *MMP13 *was not expressed despite of reports about its induction by NF-κB, JUN, or FOS. These facts indicate that the regulation of MMP and TNFα expression may be more diverse than presently known and that it still represents a relevant research target to elucidate the role of SFB in the pathophysiology of rheumatic diseases.

Concerning the formation of collagen type I fibres by COL1A1 and COL1A2 proteins following the stimulation with TGFβ1, a constitutive expression of *COL1A2 *was calculated. Based on these data, *COL1A1 *has to be regarded as the critical switch for the formation of collagen I. In contrast, the corresponding literature generally postulates a co-regulation of both genes, due to similarities in their promoters [66,67]. This difference suggests that the regulation of *COL1A1 *and *COL1A2 *may not have been fully elucidated so far possibly pointing at *COL1A1 *as a more promising target for the exploration of fibrosis.

Our analyses also show that TNFα-induced signalling predominantly results in the activation of *ETS1 *and *NFKB1*, whereas TGFβ1-related signal transduction is ultimately mediated *via *proteins of the SMAD family. Defined intervention addressing these signalling modules, alone or in combination with established therapies targeting TNFα (e.g., etanercept), may therefore improve the efficiency and outcome of current anti-rheumatic therapy [68]. Alternatively, the present results may be employed to define subpopulations of RA patients in characteristic phases of RA (active inflammatory early *versus *burnt-out/fibrotic late) and tailor anti-rheumatic treatment to the particular needs of the respective phase [69].

Both the complexity of even relatively small networks such as our ECM network and the completeness of the attribute exploration algorithm led to a large number of temporal rules. However, high support of a rule (often correlated to its simplicity) can be used as an indicator for the most meaningful hypotheses about co-regulation, mutual exclusion, and/or temporal dependencies not only between single genes, but between small sets of (functionally related) genes. The fidelity of our rules was reinforced by the comparison of simulated and observed time series data, first manually, then automatically by the attribute exploration algorithm.

Combining two well-developed algebraic, discrete and logical methods (Boolean network construction and FCA) it was possible to include human expert knowledge in all different phases (assembly of the network, adjustment to the data, and choice of relevant temporal rules), with the exception of the challenging data discretisation step. On the one hand, data discretisation is an important tool to filter out noise and to reduce complexity, but on the other hand it inevitably causes loss of information [70]. Carefully evaluating the method, we tried to keep as much important information as possible. In special cases, we consulted the original data again. A recently developed FCA-based method avoids predefined discretisation but computes an ordered set of "interval pattern structures" depending on the observed values [28]. Thus, a data set may be described without loss of information or by means of any desired granularity.

Additional method optimisations comprise strengthening the expert role on the one hand and up-scaling the network to medium size by supplementary automatisation on the other. Especially for a small set of interesting genes an interactive attribute exploration is feasible to fortify the human expert. Using this procedure for the knowledge base construction, single rules can be validated manually or by a supporting computer program, or even new experiments can be suggested. Whereas we applied a strong validation criterion requiring rules to hold for all simulated and observed transitions, the expert could also reject rules below a threshold of support and confidence in the observed context, potentially reducing noise or eliminating measurement errors.

In order to enhance computational efficiency, methods of rule selection could be integrated into the algorithm, based on association rule mining and "iceberg concept lattices" (by taking advantage of the duality between the stem base and the conceptual hierarchy (lattice) derived from a transition context as in Table 6) [71]. Manual adaptation of the network may be replaced by algorithms of network inference [26].

In summary, the adapted Boolean network model reported here for the simulation of ECM formation and degradation in rheumatic diseases may represent a powerful tool aiding computational analyses of disease-related ECM remodelling and supporting a precise design of further experiments.

## Methods

### Clinical data

#### Patients and samples

Synovial membrane samples were obtained following tissue excision upon joint replacement/synovectomy from RA and OA patients (n = 3 each; Table 5). Informed patient consent was obtained and the study was approved by the ethics committees of the respective university. RA patients were classified according to the American College of Rheumatology (ACR) criteria [72], OA patients according to the respective criteria for osteoarthritis [73].

The preparation of primary SFB from RA and OA patients was performed as previously described [65]. Briefly, the tissue samples were minced and digested with trypsin/collagenase P. The resulting single cell suspension was cultured for seven days. Non-adherent cells were removed by medium exchange. SFB were then negatively purified using Dynabeads^® ^M-450 CD14 and subsequently cultured over 4 passages in DMEM containing 100 μg/ml gentamycin, 100 μg/ml penicillin/streptomycin, 20 mM HEPES (all 100 from PAA Laboratories, Coelbe, Germany), and 10% FCS (BioWhittaker-Lonza, Basel, Switzerland).

#### Cell stimulation and isolation of total RNA

At the end of the fourth passage, the SFB were washed in serum-free DMEM and then stimulated by 10 ng/ml TGFβ1 or TNFα (PeproTech, Hamburg, Germany) in serum-free DMEM for 0, 1, 2, 4, and 12 h. At each time point, the medium was removed and the cells were harvested after treatment with trypsin (0.25% in versene; Invitrogen, Karlsruhe, Germany). After washing with PBS, they were lysed with RLT buffer (Qiagen, Hilden, Germany) and frozen at -70°C. Total RNA was isolated using the RNeasy Kit (Qiagen) according to the supplier's recommendation.

#### Microarray data analysis

Analysis of gene expression was performed using U133 Plus 2.0 RNA microarrays (Affymetrix^®^, Santa Clara, CA, USA). Labelling of RNA probes, hybridisation, and washing were carried out according to the supplier's instructions. Microarrays were analysed by laser scanning (Hewlett-Packard Gene Scanner). Background-corrected signal intensities were determined and normalised using the MAS 5.0 software (Affymetrix^®^). For this purpose, arrays were grouped according to the respective stimulus (TGFβ1 and TNFα, n = 6 each). The arrays in each group were normalised using quantile arrays in normalisation [74]. Original data from microarray analysis have been deposited in NCBI Gene Expression Omnibus [42] and are accessible through GEO series accession number GSE13837. A list of probe sets and all expression time courses are provided in additional file 4.

### Creating network and Boolean functions

For the selection of genes and proteins involved in ECM maintenance and for network generation, Boolean queries were performed in PubMed [75]. Articles were selected containing information about relevant genes expressed in SFB and involved in ECM maintenance. For information extraction, the abstracts were screened and filtered manually for statements on healthy conditions only. This knowledge-based collection yielded the set of gene candidates analysed in detail. The final gene list is presented in Table 1.

The genes were also analysed using Bibliosphere [38] and literature not extracted from PubMed was added. Subsequently, information concerning regulatory relationships was collected and transformed into short statements serving as input relations (edges) for the network building program Cytoscape, version 2.6.0 [76]. Contradictory literature information was resolved by preferring facts applying to the target cell type (human fibroblasts) and/or by comparison with experimental gene expression results from our and other microarrray data (GSE1742 and GSE2624, see additional file 5). The complete list of used statements and the respective literature basis can be found in additional file 1. In a further step, simulation results were iteratively compared to the experimental data in the present study, resulting in two adapted Boolean networks which represent hypotheses about regulatory processes initiated by TGFβ1 and TNFα.

### Data discretisation

Since we were interested in regulatory interactions, the fold-change of the expression values was more important than absolute levels. Hence, we discretised individual time series separately. The discretised data served to verify or falsify the temporal dependencies predicted from the extracted literature knowledge. For that reason, we wanted to conserve as many effects on gene expression as possible and set weak criteria for up-regulation: if the highest fold-change (i.e., the difference of log2 values) between two arbitrary time points was larger than 1, then the time profile was discretised to 0 or 1 by k-means clustering (100 iterations, vote of 25 restarts). We set the constant value 0 if: (i) the highest fold-change between two arbitrary points in a time series was less than 1; (ii) the absolute expression value was below the threshold of 100 for one probe set; or (iii) the Affymetrix detection value p indicating the reliability of the measurement exceeded 0.05. In all other cases, the constant was set to 1. Applying these criteria, also individual values were set to 0 (i.e., off) following clustering.

### Principles of simulation

Using the deterministic Boolean network, simulations were generated using an asynchronous update scheme based on the subsequent biologically-founded assumptions. In order to simulate the time courses more realistically, transcription and translation were separated, i.e., the left side of a Boolean function (output) was considered as mRNA and the right side as TF and/or stimulus (input). Unfortunately, time-resolved data for gene expression events, mRNA, or protein half-life are scarce in the literature. Therefore, time steps were selected based on general expert knowledge and comparison of literature and experimental data, if available. For example, the duration of transcription was generally set to 1 time unit, for NF-κB it was set to a doubled time period, reflecting its markedly prolonged response time before expression compared to the immediate early response transcription factors AP-1 and ETS1 [77].

In summary, we selected the time steps as follows: transcription 1 (NFKB1: 2), translation: 1, mRNA lifespan: 1, and protein lifespan: 2. Since TGFβ1 and TNFα have to be released into the extracellular medium after translation, they were assumed to take effect three time units after induction. Nevertheless, we are aware that these intervals are not absolute durations (e.g., hours), but their qualitative relationships are important. The starting conditions of the simulations were characterised by the initially observed, discretised states, and an initial state was introduced, for which the TFs were set to on. Supposing a steady state situation before starting the stimulation with TGFβ1 and TNFα, the protein levels at step 0 and 1 were defined according to that of the corresponding mRNA, and, in addition, the respective stimulating protein was set to on. The simulations were performed over twelve time units, roughly corresponding to the twelve hour duration of the gene expression experiments.

### Creation of a temporal rule knowledge base

For a detailed description of FCA mathematics and strict definitions see [78]. The sets of observed and simulated states S_obs_ and S_sim_ were characterised by the expression levels of each gene, i.e., by a subset of attributes M = E × F, with entities or genes E, and the corresponding values F = {off, on}. A state can also be considered as a tuple (f_1_, ..., f_n_) with f_i_∈ F, n = |E|.

The transitions after one time step define relations R_obs_⊆ S_obs_× S_obs_ and R_sim_⊆ S_sim_ × S_sim_ on the states. Thus, in general, multiple output states s^out^ following an input state s^in^ are possible. However, this case rarely occurred, justifying the use of a deterministic simulation procedure.

We computed the transitive closure of these relations, since we were interested in all states emerging from a given one, within the observation or simulation time. The data of all time series related to one stimulus was assembled in the "formal contexts" K_obs_ and K_sim_ which represent the basic data structure of FCA. These define relations *I *between objects (the transitions) and attributes (the discretised gene expression levels in input and output states). Accordingly, the rows in Table 6 represent transitions, expressed as tuples of input and output attributes (f_1_^in^, ..., f_n_^in^, f_1_^out^, ..., f_n_^out^).

Generally, we applied the interactive version of the attribute exploration algorithm [54] to K_sim_. It generates a minimal set of implications: A → B, A, B ⊆ M × {in, out}, which are valid in the formal context K_sim_. An implication means that if any transition has all attributes a ∈ A, then it also has all attributes b ∈ B. An expert (or, alternatively, a computer programme) is asked to validate each implication. If s/he denies, a counter-example must be provided, i.e., a new transition of the context. If s/he accepts, the implication is added to the "stem base" of the context.

As a result, a sound, complete, and non-redundant knowledge base is created, from which all implications, valid according to the semantics given by the enlarged formal context K_sim_, can be derived. In other words, the implications are valid regarding the knowledge formalised in the Boolean network and can also be checked by supplementary human expert knowledge or further literature research, e.g., for co-regulation of genes or possible or forbidden resulting states.

In this study, we compared the literature-based implications with those merely derived from the data and applied a strong criterion: implications of K_sim_ had to be valid for *all *transitions of the observed context K_obs_. This is equivalent to an exploration of the union of the two contexts, where every proposed implication is accepted. Thus, the resulting stem base was computed automatically with the Java tool Concept Explorer which supports also expert centred attribute exploration [79]. The other calculations were made with our own R [] programs (available upon request).

In the worst case, the running time of the attribute exploration algorithm depends exponentially on the size of the input data table (rows × columns) [80]. Computing the 2,713 (8,785) TGFβ1 (TNFα) rules, Concept Explorer ran 21 (30) minutes on a 2.66 GHz/2 GB computer.

### Expert analysis of transition rules

The calculated transition rules were screened manually, focussing on the appearance and the temporal behaviour of the following features: (i) constitutive *vs*. induced gene expression; (ii) co-expression *vs*. divergent expression of mediators, TFs, and target genes; (iii) expression of mediators/transcription factors *vs*. expression of target genes; (iv) regulation of target gene expression based on the expression of different transcription factors; (v) expression of individual genes *vs*. expression of their functional groups; and (vi) discrepancies to the literature. Subsequently, the extracted rules were assessed with respect to biological coherence and relevance.

## Abbreviations

BF: Boolean function; ECM: extracellular matrix; FCA: formal concept analysis; MMP: matrix metalloprotease; OA: osteoarthritis; RA: rheumatoid arthritis; SFB: synovial fibroblasts; SM: synovial membrane; TF: transcription factor.

## Authors' contributions

JW performed the bioinformatics experiments and analyses, RH participated in data analyses, RH and DP performed the stimulation experiments, and DK performed the Affymetrix microarray experiments. RG participated in design and coordination of the study and helped with bioinformatics. RWK participated in design and coordination of the study, and participated in the data analyses. UG selected ECM molecules for analyses, did the literature search, and participated in the data analyses. JW, RH and UG wrote the manuscript, supported by RWK and RG. All authors read and approved the final version of the manuscript.

## Supplementary Material

Additional file 1 **Literature used for the network construction**. Each citation corresponds to one edge in the regulatory network. Click here for file

Additional file 2 **Cytoscape import file**. Import this file into Cytoscape [] to analyse the gene regulatory network in more detail. It also includes external links for the genes and references cited to GenBank, Uniprot, and PubMed. Click here for file

Additional file 3 **Cytoscape import file**. Open this file after importing the CYS (file provided by Additional file 2) into Cytoscape [] if the layout of the CYS file cannot be displayed correctly with your Cytoscape version. Click here for file

Additional file 4 **List of probe sets used, processed microarray data, and visualisation of expression time courses for the genes analysed**. Raw data are deposited under accession number GSE13837 at . Click here for file

Additional file 5 **Processed and visualisation of GEO Data**. Data were extracted from GSE1742 (TGFβ1) and GSE2624 (TNFα) at . Click here for file

Additional file 6 **Discretised gene expression time courses**. For the discretisation method, see Results and Discussion as well as Methods sections. Click here for file

Additional file 7 **Histograms of gene expression simulation**. The simulations for TGFβ1 (blue) and TNFα (red) were run for 12 time steps (x-axis) and for each initial state derived from the patients' data separately. A simulated expression of 100% (y-axis) means that in all six cases the gene was *on*. Click here for file

Additional file 8 **List of the top 500 occurring KN rules**. Excel file containing the top 500 knowledge base rules valid for the simulations as well as for the data from stimulations with TGFβ1 and TNFα. Click here for file

Additional file 9 **Temporal rules after TGFβ1 stimulation**. Complete lists, valid for the simulation and the observed time series. Click here for file

Additional file 10 **Temporal rules after TNFα stimulation**. Complete lists, valid for the simulation and the observed time series. Click here for file

Additional file 11 **Temporal rules after TGFβ1 stimulation in PROLOG format**. E.g., for the use with the XSB Logic Programming and Deductive Database system, supporting tabled resolution . Click here for file

Additional file 12 **Temporal rules after TNFα stimulation in PROLOG format**. E.g., for the use with the XSB Logic Programming and Deductive Database system, supporting tabled resolution . Click here for file
