# Supplementary material for: Adapted Boolean network models for extracellular matrix formation
Source: BMC Syst Biol. 2009 Jul 21;3:77. doi: 10.1186/1752-0509-3-77 (PMC2734845; doi:10.1186/1752-0509-3-77)
Supplement: Additional file 1 — Literature used for the network construction. Each citation corresponds to one edge in the regulatory network. [file 1752-0509-3-77-S1.doc]

## Additional File 1 - Literature information used for ECM network creation.

Numbering correspond to the edge numbers in the ECM network shown in Figure 1; n/a: not applicable, arrow (🡪): induction, T (─╢): inhibition. Lines in grey represent facts which have not been used for network construction except for SMAD3  MMP13 which has been subsumed under SMAD4  MMP13 (No. 50).

| **No** | **Ref** | **Cell type** | **Statement** | **Information extracted** |
| --- | --- | --- | --- | --- |
| 1 | [1] | n/a | Ets1 inhibits TGFB-dependent transcription of COL1A1 and COL1A2 | ETS1 ─╢ COL1A1 |
| 2 | [1] | n/a | Ets1 strongly suppresses TGF-beta induction of collagen type I … | ETS1 ─╢ COL1A2 |
| 3 | [2] | n/a | … Ets-1 stimulate the human ets-1 promoter | ETS1 🡪 ETS1 |
| 4 | [3] | cultured mesangial cells | ... ets-1, is a transcription factor regulating the expression of … including MMP-1 … | ETS1 🡪 MMP1 |
| 5 | [3] | cultured mesangial cells | … ets-1, is a transcription factor regulating the expression of …including … MMP-3 … | ETS1 🡪 MMP3 |
| 6 | [4] | n/a | Regulation of … MMP9 … gene expression has been ascribed to Ets1 | ETS1 🡪 MMP9 |
| 7 | [5] | n/a | nfkb1 promoter is controlled by proteins of the Ets family | ETS1 🡪 NFKB |
| 8 | [6] | n/a | Ets … to two … binding sites … which are required for … TNF-alpha gene expression | ETS1 🡪 TNF |
| 9 | [7] | n/a | an AP1 binding site … involves in transcriptional regulation of COL1A1 gene | FOS 🡪 COL1A1 |
| 10 | [2] | n/a | AP-1 … stimulate the human ets-1 promoter | FOS 🡪 ETS1 |
| 11 | [8] | n/a | Fos and Jun co-operatively repress the fos promoter | FOS ─╢ FOS |
| 12 | [9] | n/a | activation of the junD promoter by JunD is abolished by c-Fos | FOS ─╢ JUND |
| 13 | [10] | synovial fibroblasts | NF-kappaB and AP-1 cooperate to mediate IL-1-induced MMP-1 transcription | FOS 🡪 MMP1 |
| 14 | [11] | synovial fibroblasts | … stromelysin gene expression can therefore be inhibited … by … agents related to AP-1… | FOS 🡪 MMP3 |
| 15 | [12] | n/a | the AP-1 (c-Fos.c-Jun)- … -binding sites … enables promoter activation. | FOS 🡪 MMP13 |
| 16 | [13] | n/a | induction of Smad7 … is mediated via induction of … AP-1 (c-Jun and c-Fos) | FOS 🡪 SMAD7 |
| 17 | [14] | dermal fibroblasts | Transcription of the TGF-beta 1 gene is … governed by the activating protein 1 (AP1) | FOS 🡪 TGFB |
| 18 | [15] | n/a | c-Fos, JunD, and c-Jun are essential for the induction of Timp-1 gene expression | FOS 🡪 TIMP1 |
| 19 | [7] | n/a | an AP1 binding site … involves in transcriptional regulation of COL1A1 gene | JUN 🡪 COL1A1 |
| 20 | [16] | n/a | overexpression of c-jun I … implicating AP-1 in the regulation of COL1A2 gene expression | JUN 🡪 COL1A2 |
| 21 | [2] | n/a | AP-1 … stimulate the human ets-1 promoter | JUN 🡪 ETS1 |
| 22 | [8] | n/a | Fos and Jun co-operatively repress the fos promoter | JUN ─╢ FOS |
| 23 | [17] | n/a | jun transcription is directly stimulated by its own gene product | JUN 🡪 JUN |
| 24 | [12] | n/a | interaction of the AP-1 factors c-Fos and c-Jun with Cbfa1 for collagenase-3 promoter activation | JUN 🡪 MMP13 |
| 25 | [11] | synovial fibroblasts | … stromelysin gene expression can therefore be inhibited … by … agents related to AP-1… | JUN 🡪 MMP3 |
| 26 | [13] | n/a | induction of Smad7 gene expression …is mediated via induction of … AP-1 (c-Jun and c-Fos) | JUN 🡪 SMAD7 |
| 27 | [15] | n/a | c-Fos, JunD, and c-Jun are essential for the induction of Timp-1 gene expression | JUN 🡪 TIMP1 |
| 28 | [6] | n/a | transcription factors … c-jun, … are also inducibly recruited to the TNF-alpha promoter | JUN 🡪 TNF |
| 29 | [18] | n/a | AP-1 … reacted with antibodies … against … c-fos, junB, and junD but not c-jun proteins | JUNB 🡪 TIMP1 |
| 30 | [7] | n/a | that JunD is required for TGF-beta -stimulated collagen synthesis | JUND 🡪 COL1A2 |
| 31 | [9] | n/a | activation of the junD promoter by JunD | JUND 🡪 JUND |
| 32 | [19] | n/a | … at both AP-1 sites contain c-Fos and Jun D proteins | JUND 🡪 MMP1 |
| 33 | [20] | epithelial cells | … in the human TGFbeta1 promoter … JunD … present … this AP-1 site | JUND 🡪 TGFB |
| 34 | [15] | n/a | c-Fos, JunD, and c-Jun are essential for the induction of Timp-1 gene expression | JUND 🡪 TIMP1 |
| 35 | [21] | osteoblasts | NF-kB binding … has a functional role in the down-regulation of COL1A1 gene transcription | NFKB ─╢ COL1A1 |
| 36 | [10, 22] | dermal fibroblasts | … NF-kB element … plays a critical role in … inhibition of COL1A2 gene transcription | NFKB ─╢ COL1A2 |
| 37 | [23] | n/a | essential role of NF-kappaB in participating in the regulation of …c-fos, .... | NFKB 🡪 FOS |
| 38 | [24] | n/a | JunB is under control of NF-kappa B | NFKB 🡪 JUNB |
| 39 | [10] | synovial fibroblasts | NF-kappaB and AP-1 cooperate to mediate IL-1-induced MMP-1 transcription | NFKB 🡪 MMP1 |
| 40 | [25] | n/a | NF-kappaB is required for cytokine upregulation of MMP-1, -3 and -9 | NFKB 🡪 MMP3 |
| 41 | [26] | n/a | NF-kappaB-regulated genes… matrix metalloproteinase 9 | NFKB 🡪 MMP9 |
| 42 | [27] | n/a | MMP13 expression … requires… nuclear factor kappaB | NFKB 🡪 MMP13 |
| 43 | [5] | n/a | NFKB is autoregulated ... activation through NF-kappa B binding sites in its promoter | NFKB 🡪 NFKB |
| 44 | [28] | HEK 293 | (TNF-alpha via) NF-kappaB activation inhibits Smad7 gene expression | NFKB ─╢ SMAD7 |
| 45 | [29] | n/a | TIMP-1 is up-regulated by … via both the IKK/NF-kappaB … | NFKB 🡪 TIMP1 |
| 46 | [30] | n/a | NF-kappa B p65 and p50 synergistically activated the TNF-alpha promoter | NFKB 🡪 TNF |
|  | [31] | n/a | Smad and AP-1 complexes function synergistically to … activation of the c-Jun promoter | SMAD3 🡪 JUN |
|  | [32] | n/a | Smad3, a TGF-beta specific Smad … induced … transcription and enhanced responses to TGF-beta | SMAD3🡪 JUNB |
| 50 | [33] | n/a | induction of MMP-13 expression by Smad3 | SMAD4 🡪 MMP13 |
|  | [34] | n/a | Smad7 promoter … DNA-binding complexes and identified Smad3, … | SMAD3 🡪 SMAD7 |
|  | [35] | n/a | have identified … tissue inhibitor of metalloproteases-1 as definite TGF-beta/Smad3 targets | SMAD3 🡪 TIMP1 |
| 47 | [36] | n/a | direct inhibition of ETS-1 transcription by SMAD4 | SMAD4 ─╢ ETS1 |
| 48 | [31] | n/a | Smad and AP-1 complexes function synergistically to … activation of the c-Jun promoter | SMAD4 🡪 JUN |
| 49 | [32] | n/a | … Smad4 induced … transcription and enhanced responses to TGF-beta | SMAD4 🡪 JUNB |
| 51 | [34] | n/a | Smad7 promoter … DNA-binding complexes and identified … Smad4 … | SMAD4 🡪 SMAD7 |
| 52 | [37] | n/a | amplification of TIMP-1 is mediated by … Smad-3 and Smad-4 proteins | SMAD4 🡪 TIMP1 |
| 53 | [7] | lung fibroblasts | TGF-beta-induced COL1A1 mRNA level | TGFB 🡪 COL1A1 |
| 54 | [7] | dermal fibroblasts | TGF-beta -stimulated COL1A2 transcription | TGFB 🡪 COL1A2 |
| 55 | [38] | n/a | TGF-beta1 addition causes an immediate and transient induction of c-fos … | TGFB 🡪 FOS |
| 56 | [7] | dermal fibroblasts | TGF-beta induces the level of Jun-B | TGFB 🡪 JUNB |
| 57 | [39] | human KMST fibroblasts | TGF-beta - strongly enhanced the expression of junD | TGFB 🡪 JUND |
| 58 | [40] | synovial fibroblasts | TGF-beta could antagonize phorbol ester induction of MMP-1 in synovial fibroblasts | TGFB ─╢MMP1 |
| 59 | [41] | synovial fibroblasts | … but it facilitates the TGF-beta-induced expression of MMP-3 significantly | TGFB 🡪 MMP3 |
| 60 | [39] | human KMST fibroblasts | TGF-beta1 … assumed to be inhibitory for MMPs, strongly induces collagenase-3 | TGFB 🡪 MMP13 |
| 61 | [32] | n/a | NF-kappaB sites … mediate transcriptional activation by TGF-beta … mediated by Smad members | TGFB 🡪 NFKB |
| 62 | [42]; [43] | n/a | TGF-beta -> runx2: PMID: 15084595. runX --> Smad4 | TGFB 🡪 SMAD4 |
| 63 | [44] | n/a | TGF-beta rapidly induces expression of Smad7 mRNA | TGFB 🡪 SMAD7 |
| 64 | [45] | human lung fibroblasts | … TGF-betas significantly decreased MMP-1 and increased TIMP-1, IL-6 and collagen type I | TGFB 🡪 TIMP1 |
| 65 | [7] | rat hepatic stellate cells | TNF-alpha … inhibits COL1A1 gene expression | TNF ─╢ COL1A1 |
| 66 | [22] | n/a | … TNF-alpha inhibitory effect on COL1A2 transcription | TNF ─╢ COL1A2 |
| 67 | [1] | synovial fibroblasts | … synovial fibroblasts, the most potent inducers of Ets1 are … such as TNF-alpha | TNF 🡪 ETS1 |
| 68 | [46] | murine embryo fibroblasts | TNF caused increased expression of c-Jun, JunB, JunD, c-Fos … | TNF 🡪 FOS |
| 69 | [10] | synovial fibroblasts | IL-1 is superior to TNF at inducing c-Jun synthesis … | TNF 🡪 JUN |
| 70 | [47] | n/a | c-Jun and JunB, both induced by TNF-alpha | TNF 🡪 JUNB |
| 71 | [46] | murine embryo fibroblasts | TNF caused increased expression of c-Jun, JunB, JunD, c-Fos … | TNF 🡪 JUND |
| 72 | [48] | dermal fibroblasts | MMP-1 and TIMP-1 mRNA expression … increased with …. TNF-alpha treatment | TNF 🡪 MMP1 |
| 73 | [49] | synovial fibroblasts | … TNF-alpha… mediate induction of matrix metalloproteinases, MMP3 … | TNF 🡪 MMP3 |
| 74 | [50] | bronchial fibroblasts | these data demonstrate that TNF-alpha increases MMP-9 | TNF 🡪 MMP9 |
| 75 | [49] | synovial fibroblasts | … TNF-alpha… mediate induction of matrix metalloproteinases, … MMP13 | TNF 🡪 MMP13 |
| 76 | [10] | synovial fibroblasts | … and TNF activate NF-kappaB in these cells | TNF 🡪 NFKB |
| 77 | [28] | HEK 293 | (TNF-alpha via) NF-kappaB activation inhibits Smad7 gene expression | TNF ─╢ SMAD7 |
| 78 | [48] | dermal fibroblasts | MMP-1 and TIMP-1 were markedly increased with IL-6 and TNF-alpha treatment | TNF 🡪 TIMP1 |
| 79 | [46] | n/a | TNF-stimulated TNF-alpha expression … | TNF 🡪 TNF |

# References

1. Czuwara-Ladykowska J, Sementchenko VI, Watson DK, Trojanowska M: **Ets1 is an effector of the transforming growth factor beta (TGF-beta ) signaling pathway and an antagonist of the profibrotic effects of TGF-beta.** *J Biol Chem* 2002, **277:**20399-20408.

2. Majerus MA, Bibollet-Ruche F, Telliez JB, Wasylyk B, Bailleul B: **Serum, AP-1 and Ets-1 stimulate the human ets-1 promoter.** *Nucleic Acids Res* 1992, **20:**2699-2703.

3. Mizui M, Isaka Y, Takabatake Y, Sato Y, Kawachi H, Shimizu F, Takahara S, Ito T, Imai E: **Transcription factor Ets-1 is essential for mesangial matrix remodeling.** *Kidney Int* 2006, **70:**298-305.

4. Dittmer J: **The biology of the Ets1 proto-oncogene.** *Mol Cancer* 2003, **2:**29.

5. Lambert PF, Ludford-Menting MJ, Deacon NJ, Kola I, Doherty RR: **The nfkb1 promoter is controlled by proteins of the Ets family.** *Mol Biol Cell* 1997, **8:**313-323.

6. Tsai EY, Falvo JV, Tsytsykova AV, Barczak AK, Reimold AM, Glimcher LH, Fenton MJ, Gordon DC, Dunn IF, Goldfeld AE: **A lipopolysaccharide-specific enhancer complex involving Ets, Elk-1, Sp1, and CREB binding protein and p300 is recruited to the tumor necrosis factor alpha promoter in vivo.** *Mol Cell Biol* 2000, **20:**6084-6094.

7. Ghosh AK: **Factors involved in the regulation of type I collagen gene expression: implication in fibrosis.** *Exp Biol Med (Maywood)* 2002, **227:**301-314.

8. Konig H, Ponta H, Rahmsdorf U, Buscher M, Schonthal A, Rahmsdorf HJ, Herrlich P: **Autoregulation of fos: the dyad symmetry element as the major target of repression.** *Embo J* 1989, **8:**2559-2566.

9. Berger I, Shaul Y: **c-Fos antagonizes the junD gene positive autoregulatory loop; a novel c-Fos role in promoter switching.** *Gene* 1998, **211:**375-382.

10. Barchowsky A, Frleta D, Vincenti MP: **Integration of the NF-kappaB and mitogen-activated protein kinase/AP-1 pathways at the collagenase-1 promoter: divergence of IL-1 and TNF-dependent signal transduction in rabbit primary synovial fibroblasts.** *Cytokine* 2000, **12:**1469-1479.

11. Morin I, Li WQ, Su S, Ahmad M, Zafarullah M: **Induction of stromelysin gene expression by tumor necrosis factor alpha is inhibited by dexamethasone, salicylate, and N-acetylcysteine in synovial fibroblasts.** *J Pharmacol Exp Ther* 1999, **289:**1634-1640.

12. D'Alonzo RC, Selvamurugan N, Karsenty G, Partridge NC: **Physical interaction of the activator protein-1 factors c-Fos and c-Jun with Cbfa1 for collagenase-3 promoter activation.** *J Biol Chem* 2002, **277:**816-822.

13. Quan T, He T, Voorhees JJ, Fisher GJ: **Ultraviolet irradiation induces Smad7 via induction of transcription factor AP-1 in human skin fibroblasts.** *J Biol Chem* 2005, **280:**8079-8085.

14. Varedi M, Ghahary A, Scott PG, Tredget EE: **Cytoskeleton regulates expression of genes for transforming growth factor-beta 1 and extracellular matrix proteins in dermal fibroblasts.** *J Cell Physiol* 1997, **172:**192-199.

15. Hall MC, Young DA, Waters JG, Rowan AD, Chantry A, Edwards DR, Clark IM: **The comparative role of activator protein 1 and Smad factors in the regulation of Timp-1 and MMP-1 gene expression by transforming growth factor-beta 1.** *J Biol Chem* 2003, **278:**10304-10313.

16. Chung KY, Agarwal A, Uitto J, Mauviel A: **An AP-1 binding sequence is essential for regulation of the human alpha2(I) collagen (COL1A2) promoter activity by transforming growth factor-beta.** *J Biol Chem* 1996, **271:**3272-3278.

17. Angel P, Hattori K, Smeal T, Karin M: **The jun proto-oncogene is positively autoregulated by its product, Jun/AP-1.** *Cell* 1988, **55:**875-885.

18. Ulisse S, Farina AR, Piersanti D, Tiberio A, Cappabianca L, D'Orazi G, Jannini EA, Malykh O, Stetler-Stevenson WG, D'Armiento M: **Follicle-stimulating hormone increases the expression of tissue inhibitors of metalloproteinases TIMP-1 and TIMP-2 and induces TIMP-1 AP-1 site binding complex(es) in prepubertal rat Sertoli cells.** *Endocrinology* 1994, **135:**2479-2487.

19. White LA, Brinckerhoff CE: **Two activator protein-1 elements in the matrix metalloproteinase-1 promoter have different effects on transcription and bind Jun D, c-Fos, and Fra-2.** *Matrix Biol* 1995, **14:**715-725.

20. Liu G, Ding W, Liu X, Mulder KM: **c-Fos is required for TGFbeta1 production and the associated paracrine migratory effects of human colon carcinoma cells.** *Mol Carcinog* 2006, **45:**582-593.

21. Roebuck KA, Vermes C, Carpenter LR, Fritz EA, Narayanan R, Glant TT: **Down-regulation of procollagen alpha1[I]] messenger RNA by titanium particles correlates with nuclear factor kappaB (NF-kappaB) activation and increased rel A and NF-kappaB1 binding to the collagen promoter.** *J Bone Miner Res* 2001, **16:**501-510.

22. Kouba DJ, Chung KY, Nishiyama T, Vindevoghel L, Kon A, Klement JF, Uitto J, Mauviel A: **Nuclear factor-kappa B mediates TNF-alpha inhibitory effect on alpha 2(I) collagen (COL1A2) gene transcription in human dermal fibroblasts.** *J Immunol* 1999, **162:**4226-4234.

23. Fujioka S, Niu J, Schmidt C, Sclabas GM, Peng B, Uwagawa T, Li Z, Evans DB, Abbruzzese JL, Chiao PJ: **NF-kappaB and AP-1 connection: mechanism of NF-kappaB-dependent regulation of AP-1 activity.** *Mol Cell Biol* 2004, **24:**7806-7819.

24. Mathas S, Hinz M, Anagnostopoulos I, Krappmann D, Lietz A, Jundt F, Bommert K, Mechta-Grigoriou F, Stein H, Dorken B, Scheidereit C: **Aberrantly expressed c-Jun and JunB are a hallmark of Hodgkin lymphoma cells, stimulate proliferation and synergize with NF-kappa B.** *Embo J* 2002, **21:**4104-4113.

25. Bond M, Chase AJ, Baker AH, Newby AC: **Inhibition of transcription factor NF-kappaB reduces matrix metalloproteinase-1, -3 and -9 production by vascular smooth muscle cells.** *Cardiovasc Res* 2001, **50:**556-565.

26. Shukla S, Gupta S: **Suppression of constitutive and tumor necrosis factor alpha-induced nuclear factor (NF)-kappaB activation and induction of apoptosis by apigenin in human prostate carcinoma PC-3 cells: correlation with down-regulation of NF-kappaB-responsive genes.** *Clin Cancer Res* 2004, **10:**3169-3178.

27. Mengshol JA, Vincenti MP, Coon CI, Barchowsky A, Brinckerhoff CE: **Interleukin-1 induction of collagenase 3 (matrix metalloproteinase 13) gene expression in chondrocytes requires p38, c-Jun N-terminal kinase, and nuclear factor kappaB: differential regulation of collagenase 1 and collagenase 3.** *Arthritis Rheum* 2000, **43:**801-811.

28. Nagarajan RP, Chen F, Li W, Vig E, Harrington MA, Nakshatri H, Chen Y: **Repression of transforming-growth-factor-beta-mediated transcription by nuclear factor kappaB.** *Biochem J* 2000, **348 Pt 3:**591-596.

29. Wilczynska KM, Gopalan SM, Bugno M, Kasza A, Konik BS, Bryan L, Wright S, Griswold-Prenner I, Kordula T: **A novel mechanism of tissue inhibitor of metalloproteinases-1 activation by interleukin-1 in primary human astrocytes.** *J Biol Chem* 2006, **281:**34955-34964.

30. Liu H, Sidiropoulos P, Song G, Pagliari LJ, Birrer MJ, Stein B, Anrather J, Pope RM: **TNF-alpha gene expression in macrophages: regulation by NF-kappa B is independent of c-Jun or C/EBP beta.** *J Immunol* 2000, **164:**4277-4285.

31. Wong C, Rougier-Chapman EM, Frederick JP, Datto MB, Liberati NT, Li JM, Wang XF: **Smad3-Smad4 and AP-1 complexes synergize in transcriptional activation of the c-Jun promoter by transforming growth factor beta.** *Mol Cell Biol* 1999, **19:**1821-1830.

32. Lopez-Rovira T, Chalaux E, Rosa JL, Bartrons R, Ventura F: **Interaction and functional cooperation of NF-kappa B with Smads. Transcriptional regulation of the junB promoter.** *J Biol Chem* 2000, **275:**28937-28946.

33. Leivonen SK, Chantry A, Hakkinen L, Han J, Kahari VM: **Smad3 mediates transforming growth factor-beta-induced collagenase-3 (matrix metalloproteinase-13) expression in human gingival fibroblasts. Evidence for cross-talk between Smad3 and p38 signaling pathways.** *J Biol Chem* 2002, **277:**46338-46346.

34. Stopa M, Anhuf D, Terstegen L, Gatsios P, Gressner AM, Dooley S: **Participation of Smad2, Smad3, and Smad4 in transforming growth factor beta (TGF-beta)-induced activation of Smad7. THE TGF-beta response element of the promoter requires functional Smad binding element and E-box sequences for transcriptional regulation.** *J Biol Chem* 2000, **275:**29308-29317.

35. Verrecchia F, Chu ML, Mauviel A: **Identification of novel TGF-beta /Smad gene targets in dermal fibroblasts using a combined cDNA microarray/promoter transactivation approach.** *J Biol Chem* 2001, **276:**17058-17062.

36. Duda DG, Sunamura M, Lefter LP, Furukawa T, Yokoyama T, Yatsuoka T, Abe T, Inoue H, Motoi F, Egawa S, et al: **Restoration of SMAD4 by gene therapy reverses the invasive phenotype in pancreatic adenocarcinoma cells.** *Oncogene* 2003, **22:**6857-6864.

37. Akool el S, Doller A, Muller R, Gutwein P, Xin C, Huwiler A, Pfeilschifter J, Eberhardt W: **Nitric oxide induces TIMP-1 expression by activating the transforming growth factor beta-Smad signaling pathway.** *J Biol Chem* 2005, **280:**39403-39416.

38. Osaki M, Tsukazaki T, Yonekura A, Miyazaki Y, Iwasaki K, Shindo H, Yamashita S: **Regulation of c-fos gene induction and mitogenic effect of transforming growth factor-beta1 in rat articular chondrocyte.** *Endocr J* 1999, **46:**253-261.

39. Uria JA, Jimenez MG, Balbin M, Freije JM, Lopez-Otin C: **Differential effects of transforming growth factor-beta on the expression of collagenase-1 and collagenase-3 in human fibroblasts.** *J Biol Chem* 1998, **273:**9769-9777.

40. White LA, Mitchell TI, Brinckerhoff CE: **Transforming growth factor beta inhibitory element in the rabbit matrix metalloproteinase-1 (collagenase-1) gene functions as a repressor of constitutive transcription.** *Biochim Biophys Acta* 2000, **1490:**259-268.

41. Hoberg M, Rudert M, Pap T, Klein G, Gay S, Aicher WK: **Attachment to laminin-111 facilitates transforming growth factor beta-induced expression of matrix metalloproteinase-3 in synovial fibroblasts.** *Ann Rheum Dis* 2007, **66:**446-451.

42. Selvamurugan N, Kwok S, Partridge NC: **Smad3 interacts with JunB and Cbfa1/Runx2 for transforming growth factor-beta1-stimulated collagenase-3 expression in human breast cancer cells.** *J Biol Chem* 2004, **279:**27764-27773.

43. Young DW, Hassan MQ, Yang XQ, Galindo M, Javed A, Zaidi SK, Furcinitti P, Lapointe D, Montecino M, Lian JB, et al: **Mitotic retention of gene expression patterns by the cell fate-determining transcription factor Runx2.** *Proc Natl Acad Sci U S A* 2007, **104:**3189-3194.

44. Nakao A, Afrakhte M, Moren A, Nakayama T, Christian JL, Heuchel R, Itoh S, Kawabata M, Heldin NE, Heldin CH, ten Dijke P: **Identification of Smad7, a TGFbeta-inducible antagonist of TGF-beta signalling.** *Nature* 1997, **389:**631-635.

45. Papakonstantinou E, Aletras AJ, Roth M, Tamm M, Karakiulakis G: **Hypoxia modulates the effects of transforming growth factor-beta isoforms on matrix-formation by primary human lung fibroblasts.** *Cytokine* 2003, **24:**25-35.

46. Ventura JJ, Kennedy NJ, Lamb JA, Flavell RA, Davis RJ: **c-Jun NH(2)-terminal kinase is essential for the regulation of AP-1 by tumor necrosis factor.** *Mol Cell Biol* 2003, **23:**2871-2882.

47. Verrecchia F, Pessah M, Atfi A, Mauviel A: **Tumor necrosis factor-alpha inhibits transforming growth factor-beta /Smad signaling in human dermal fibroblasts via AP-1 activation.** *J Biol Chem* 2000, **275:**30226-30231.

48. Dasu MR, Barrow RE, Spies M, Herndon DN: **Matrix metalloproteinase expression in cytokine stimulated human dermal fibroblasts.** *Burns* 2003, **29:**527-531.

49. Sweeney SE, Hammaker D, Boyle DL, Firestein GS: **Regulation of c-Jun phosphorylation by the I kappa B kinase-epsilon complex in fibroblast-like synoviocytes.** *J Immunol* 2005, **174:**6424-6430.

50. Nakamura Y, Esnault S, Maeda T, Kelly EA, Malter JS, Jarjour NN: **Ets-1 regulates TNF-alpha-induced matrix metalloproteinase-9 and tenascin expression in primary bronchial fibroblasts.** *J Immunol* 2004, **172:**1945-1952.
